# Predictive value of inflammatory factors on the efficacy of adjuvant Dexamethasone in the treatment of refractory purulent meningitis among pediatric patients

**DOI:** 10.5937/jomb0-37618

**Published:** 2024-06-15

**Authors:** XiaoMei Zhong, QingJun Niu, XunLing Yuan

**Affiliations:** 1 Ganzhou People's Hospital, Department of Paediatrics, Ganzhou City, China; 2 Huaian Hospital, Department of Paediatrics, Huaian City, China; 3 Heilongjiang Provincial Hospital, Department of Paediatrics, Harbin City, China

**Keywords:** inflammation factors, Dexamethasone, pediatric refractory purulent meningitis, faktori zapaljenja, deksametazon, pedijatrijski refraktorni gnojni meningitis

## Abstract

**Background:**

The aim of this study was to figure out the predictive value of inflammatory factors on the efficacy of Dexamethasone adjuvant therapy for refractory purulent meningitis in children.

**Methods:**

In, this study, a regression analysis method was employed to select a sample of 38 children with refractory purulent meningitis, 40 children with purulent meningitis, and 40 healthy children who visited to Ganzhou People's Hospital for physical These participants were then assigned to the Dexamethasone, standard care and the control groups. The inflammatory factors in the three groups were compared, and a multivariate Logisitic regression was analysis was conducted to examine the predictive indicators and efficacy of Dexamethasone treatment in children with refractory purulent meningitis.

**Results:**

The levels of CRP, TNF-a, IL-6, PCT and IL-1 were found to be significantly higher in the Dexamethasone group to both the standard care and the control (P < 0.05). Through multivariate Logisitic regression analysis, it was determined that CRP, TNF-a, IL-6, PCT, and IL-1 were reliable predictors of the efficacy of Dexamethasone treatment in children with refractory purulent meningitis. These biomarkers demonstrated good predictive performance, with CRP and IL-1 showing superior predictive performance.

**Conclusions:**

Inflammatory factors have a certain predictive value for the efficacy of Dexamethasone adjuvant therapy for refractory purulent meningitis in pediatric patients.

## Abstract (sr)

**Uvod:**

Cilj ovog istraživanja bio je da se utvrdi prediktivna vrednost inflamatornih faktora na efikasnost adjuvantne terapije deksametazonom za refraktorni gnojni meningitis kod dece.

**Metode:**

U ovoj studiji korišćena je metoda regresione analize za odabir uzorka od 38 dece sa refraktornim gnojnim meningitisom, 40 dece sa gnojnim meningitisom i 40 zdrave dece koja su posetila Narodnu bolnicu okruga Kingtian u gradu Lišui radi fizičke bolesti. Ovi učesnici su bili zatim dodeljen deksametazonu, standardnoj nezi i kontrolnim grupama. Upoređeni su inflamatorni faktori u tri grupe i sprovedena je multivarijantna analiza logističke regresije da bi se ispitali prediktivni pokazatelji i efikasnost lečenja deksametazonom kod dece sa refraktornim gnojnim meningitisom.

**Rezultati:**

Utvrđeno je da su nivoi CRP, TNF-a, IL-6, PCT i IL1 značajno viši u grupi koja je primala deksametazon u odnosu na standardnu negu i kontrolu (P < 0,05). Multi - varijantnom logističkom regresionom analizom utvrđeno je da su CRP, TNF-a, IL-6, PCT i IL-1 pouzdani prediktori efikasnosti lečenja deksametazonom kod dece sa refraktornim gnojnim meningitisom. Ovi biomarkeri su pokazali dobre prediktivne performanse, pri čemu su CRP i IL-1 pokazali superiorne prediktivne performanse.

**Zaključak:**

Inflamatorni faktori imaju određenu prediktivnu vrednost za efikasnost adjuvantne terapije deksametazonom za refraktorni gnojni meningitis kod pedijatrijskih pacijenata.

## Introduction

Purulent meningitis is central nervous system common purulent infection, and its main clinical symptom is persistently elevating intracranial pressure, meningeal irritation sign, fever, convulsions, etc. Most often take place in neonates and children. It has some characteristics like rapid onset, rapid development and serious illness. The disability rate associated with purulent meningitis ranges from 20% to 50%, while the fatality rate can reach 10% to 15%, thereby posing a significant threat to the overall health of children [1]. At present, a sufficient and sufficient dose of antibiotics should be used as soon as possible to treat purulent meningitis. With the continuous maturation of antibiotics, the treatment rate of the disease is gradually elevated, and the disability rate is decreasing. However, some children with purulent meningitis are not satisfied with the curative effect of conventional antibiotic treatment, and the symptoms such as fever and convulsions recur, and have a high disability rate and mortality rate. This particular group of children is strongly associated with the specific pathogen causing the infection, and is clinically categorized as children with refractory purulent meningitis, which warrants attention from healthcare professionals. In recent years, inflammatory factors have been extensively employed in the clinical diagnosis, efficacy and prognosis evaluation of refractory purulent meningitis in children. C-reactive protein (CRP), tumor necrosis factor-α (TNF-α), interleukin-1β (IL-1β), interleukin-6 (IL-6), procalcitonin (PCT) are considered being closely implicated in the presence and progression of purulent meningitis [2]. Dexamethasone has been shown to have significant anti-inflammatory effects in children with refractory purulent meningitis [3]. There is limited research on the predictive value of inflammatory factors of children with refractory purulent meningitis. Based on this, this study aims to investigate the potential of inflammatory factors in predicting the effectiveness of dexamethasone in the treatment of refractory purulent meningitis in children.

## Materials and methods

### General information

From January 2020 to December 2021, 38 children with refractory purulent meningitis, 40 children with purulent meningitis, and 40 healthy children who were admitted to Ganzhou People's Hospital for physical examination during the same period were selected as the research subjects and assigned into the dexamethasone group, standard care and control groups in turn by regression analysis. This study was approved by the Institutional Review Committee of Ganzhou People's Hospital (Approval Number: 2019GZ1106). The experimental consisted of 25 males and 13 females, at the age of 1–6 years, with an average age of (3.48±1.04) years. The control covered 24 males and 16 females, at the age of 1–6 years, with an average age of (3.41±1.01) years. The healthy contained 23 males and 17 females, at the age of 1–6 years, with an average age of (3.36±1.11) years. No clear difference exhibited in general data such as gender and age among the three groups (*P* = 0.936 *P* = 0.873).

### Diagnosis, inclusion and exclusion criteria

The symptoms and results of the subjects meet the diagnostic criteria for purulent meningitis (Supplementary Table I). Diagnostic criteria: in the light of the diagnostic criteria of refractory purulent meningitis in children in *Zhu Futang Practical Pediatrics* [4]: pediatric patients who meet one or more of the following criteria are classified as refractory purulent meningitis: (i) the main clinical symptoms are acute fever, convulsions, depressed mood, lethargy, irritability, etc. (ii) with bregma bulge, meningeal irritation sign and other signs; (iii) Abnormal tabular brain parenchymal areas on CT or MRI of the head; (iv) Accompanying persistent complications such as subdural effusion, ependymitis, and hydrocephalus; (v) Sequela during death or late follow- up period, such as secondary epilepsy, cranial nerve injury, and psychomotor delay; (vi) After one week of conventional treatment (penicillin ceftriaxone and cefotaxime), there are still symptoms of fever or other recurrent purulent meningitis. (vii) Recurrent purulent intracranial infection of unknown origin.

Inclusion criteria: complete clinical data; no previous treatment with dexamethasone; aging 1–7 years; all children's guardians gave informed consent and signed an informed consent form.

Exclusion criteria: combined with other organ dysfunction symptoms; immunodeficiency, systemic infection; septic shock; fungal meningitis, tuberculous meningitis and other non-bacterial central nervous system infections; intracranial hemorrhage, craniocerebral trauma, brain tumor and other diseases; the children whose guardians are unwilling to participate in this research.

## Methods

The control was given infusion of 20% mannitol 125 mL, once a day, to lower elevated intracranial pressure and to improve cerebral perfusion pressure. In addition to conventional antibiotics (penicillin and cephalosporins). If the use of penicillin and cephalosporin antibacterial therapy is ineffective, the children should be given intravenous infusion of meropenem (manufacturer: sumitomo pharmaceutical (Suzhou) Co., LTD., batch no. 20161123) 40 mg·kg, once a day.

The experimental was given dexamethasone (manufacturer: Tianjin Jinyao Pharmaceutical Co., Ltd.; batch number: 12091821) on basis of the treatment of the standard care group. The first intravenous injection was 10 mg/m^2^, and then dexamethasone 15 mg/m^2^ was intravenously dripped to the 5% glucose injection. The above treatments were all continued for 7 d.

### Observation indicators

Correlation analysis between general clinical indicators (gender age disease duration APACHE II score clinical symptoms) and refractory purulent meningitis in children. The inflammation factors in the three groups were compared, the predictors of the efficacy of dexamethasone treatment in children with refractory purulent meningitis, and the predictiveefficacy of CRP, TNF-α, IL-6, PCT, IL-1β on dexamethasone treatment in children with refractory purulent meningitis was observed.

After 7 days of treatment, 3 mL of fasting venous blood was drawn from all subjects, and all samples are processed within 2–5 hours, and the supernatant was collected after centrifugation at 3000 r/min (centrifugation radius 13.5 cm). Inflammation factors: CRP, TNF-α, IL-6, PCT and IL-1β were determined by ELISA. Reference ranges of individual inflammatory parameters. CRP<8 mg/L, TNF-α<50 ng/L, IL-6 <10 pg/mL, PCT<0.5 μg/L IL-1β<15 ng/L.

### Statistical methods

SPSS 21.0 software was employed to analyze the data. The measurement data conforming to the normal distribution were clarified as x̄±s; The overall comparison of the data in each group was by one-way analysis of variance, and the pairwise comparison of the data between groups and within the group was by the LSD method; the count data were illustrated by the rate (%), and the chi-square 2 test was used. The parameters with statistically significant differences in the univariate analysis were included in the multivariate logistic regression model for analysis. Multivariate logisitic regression was applied to analyze the predictors of the efficacy of dexamethasone treatment in children with refractory purulent meningitis. Receiver operating characteristic (ROC) curve was drawn to evaluate the predictive value of serum CRP, TNF-α, IL-6, PCT, and IL-1β on the efficacy of dexamethasone treatment in children with refractory purulent meningitis. Good predictive performance has an AUC of 0.75–1.00. *P* < 0.05 emphasized obvious statistical meaning.

## Results

### Comparison of general data of three groups

No clear difference was exhibited in gender or age in the general data of the three groups (*P* =0.936, *P* = 0.873); No distinct difference was presented in clinical symptoms between the experimental and the control (*P* = 0.905); The course of disease and APACHE score in the experimental were higher than the control group (*P* < 0.05, Table 1).

**Table 1:** Comparison of general data of the three groups. Note: #P < 0.05 vs. the control; *P < 0.05 vs. the healthy.

Items	Damethasone group (38)	Standard care group (40)	Control group (40)
Gender (male/female, n)	25/13	24/16	23/17
Age (years)	3.48±1.04	3.41±1.01	3.36±1.11
Disease duration (d)	16.39±3.12	10.32±3.09	-
APACHE II (points)	17.29±2.52	12.24±2.49	-
Clinical symptoms (n)			
Headache	8	7	-
Convulsions	15	13	-
Vomit	12	11	-
Coma	3	3	-
Meningeal irritation sign	7	6	-

### Comparison of inflammation factors among the three groups

Next, we compared the differences in inflammatory factors between groups. And we found that CRP, TNF-α, IL-6, PCT and IL-1β were higher in the experimental vs. the control and the healthy, and in the control vs. the healthy, with clear difference (*P* < 0.05, Table 2).

**Table 2:** Comparison of inflammation factors levels among the three groups (x̄±s). Note: #P < 0.05 vs. the control; *P < 0.05 vs. the healthy.

Indexes	Groups (n)	CRP (mg/L)	TNF-α (ng/L)	IL-6 (pg/mL)	PCT (μg/L)	IL-1 (ng/L)
Cerebrospinal <br>fluid	Damethasone <br>group (38)	14.28±1.29#*	69.29±5.98#*	15.29±1.87#*	10.76±0.07#*	118.98±8.72#*
	Standard care <br>group (40)	10.23±1.18*	60.98±5.01*	15.19±1.76*	7.58±0.03*	110.87±7.65*
	Control Group (40)	1.07±0.13	11.19±1.65	2.87±1.09	0.13±0.01	2.35±0.32

### Multivariate logistic regression analysis of predictive indicators of dexamethasone treatment effect in children with refractory purulent meningitis

The variables examined in the univariate analysis, including the course of disease, APACHE II score, CRP, TNF-α, IL-6, PCT, and IL-1β levels. The dependent variable of interest was the efficacy of dexamethasone treatment in children with refractory purulent meningitis. Subsequently, a multivariate logistic regression analysis was performed, revealing that CRP, TNF-α, IL-6, PCT, and IL-1β were significant predictors of the efficacy of dexamethasone treatment in this population (Table 3).

**Table 3:** Multifactor logisitic regression analysis of predictors of therapeutic effect after dexamethasone treatment in children with refractory purulent meningitis.

Variables	β	S.E.	Wald value	OR (95% CI)	P-value
Disease duration	0.198	0.298	1.812	1.103 (0.641–1.567)	0.119
APACHE score	0.108	0.265	2.054	1.209 (0.563–1.921)	0.087
CRP	1.138	0.579	3.365	3.127 (1.865.4.387)	0.000
TNF-α	0.768	0.554	2.389	2.154 (1.053–3.219)	0.004
IL-6	0.789	0.609	2.107	2.208 (1.002–3.128)	0.019
PCT	0.917	0.673	3.321	2.901 (1.038, 4.107)	0.036
IL-1	1.257	0.532	4.769	3.987 (1.319, 5.651)	0.006

### Predictive efficacy of CRP, TNF-α, IL-6, PCT, IL- 1β on the efficacy of dexamethasone treatment in children with refractory purulent meningitis

**Figure 1:**
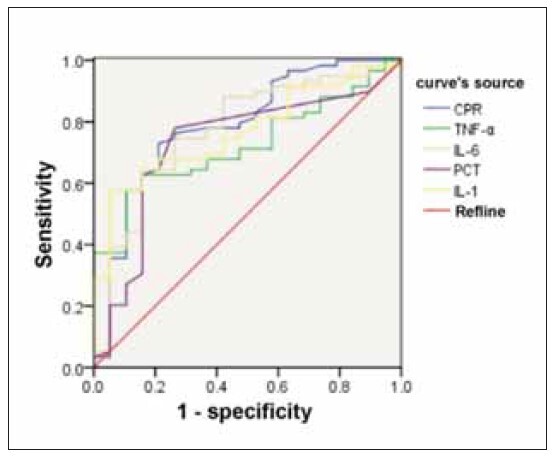
Predictive efficacy of CRP, TNF-α, IL-6, PCT and IL-1β in dexamethasone treatment of refractory purulent meningitis in children.

The AUCs values for CRP, TNF-α, IL-6, PCT, and IL-1β in predicting the efficacy of dexamethasone in children with refractory purulent meningitis were 0.877, 0.798, 0.765, 0.736, and 0.817, respectively. These values indicate good prediction performance. Notably CRP and IL-1β exhibited superior prediction performance, as demonstrated in Table 4 and Figure 1.

**Table 4:** Predictive efficacy of CRP, TNF-α, IL-6, PCT and IL-1β in children with refractory purulent meningitis treated with dexamethasone.

Items	AUC (95% CI)	The <br>cut-off value	P-value	Specificity	Sensitivity
CRP	0.877 (0.789, 0.973)	13.35	＜0.001	0.812	0.839
TNF-α	0.798 (0.692, 0.846)	67.82	＜0.001	0.756	0.769
IL-6	0.765 (0.654, 0.842)	14.89	＜0.001	0.701	0.737
PCT	0.736 (0.653, 0.834)	10.78	＜0.001	0.689	0.701
IL-1	0.817 (0.756, 0.932)	116.32	＜0.001	0.762	0.789

## Discussion

Purulent meningitis, a familiar condition in the field of pediatrics, primarily arises from infectious triggered by pathogenic bacteria, including *Escherichia coli*, *Klebsiella pneumoniae*, and *Staphylococcus aureus* invading the pia mater [4]. This invasion can result in detrimental effects on the nervous system of children. In the absence of prompt and efficacious intervention, the condition may give rise to neurological sequelae, such as sensorineural deafness, cognitive impairment, and residual motor dysfunctions, and potentially culminate in fatality among pediatric patients [5]. However, despite timely treatment being administered to certain children, the presence of the blood-cerebrospinal fluid barrier hinders the accurate identification of cerebrospinal fluid pathogens., resulting in empirically blind antibacterial, and untargeted and standardized selection of antibacterial drugs, resulting in refractory purulent meningitis in children. Pediatric refractory purulent meningitis has a high morbidity and mortality rate, necessitating the use of reliable indicators to assess its effectiveness in children with this condition. Dexamethasone, a commonly employed corticosteroid in clinical settings, exhibits favorable pharmacokinetic properties. The pharmacological effects of dexamethasone encompass antiviral, anti-inflammatory, and anti-allergic properties. It exerts its anti-inflammatory effect by inhibiting the synthesis and release of inflammatory factors in monocytes and T lymphocytes, as well as suppressing the aggregation of hemameba and macro phages at the site of inflammation. Furthermore, dexamethasone hinders refrain the formation of chemical transmitters during the inflammatory and metamorphic. At present, dexamethasone is frequently utilized in the clinical treatment of refractory purulent meningitis in children, yielding satisfactory outcomes [6] [7] [8]. Nowadays, various methods such as clinical symptoms and signs, Glasgow coma score, imaging examination and other indicators are frequently applied to evaluate the clinical efficacy of children with refractory purulent meningitis. However, there remains a dearth of objective and quantifiable evaluation indicators. This is particularly challenging in young children who lack typical clinical symptoms, making it difficult to determine their mental awareness and evaluate the extent of inflammation and the efficacy of drug treatment in a timely manner.

Previous research indicates that following infection with pathogenic bacteria, purulent meningitis stimulates the brain tissue to produce a variety of cytokines, which can impact the immune function of children and the efficacy [9] [10]. Several studies have confirmed CRP, TNF-α, IL-6, PCT, IL-1β, and other pro-inflammatory factors are elevated in the cerebrospinal fluid of children with refractory purulent meningitis, demonstrating significant diagnostic value [11] [12]. For example, he research conducted by Freer *et al.* [13] points out IL-6 and TNF-α are elevated in young children with purulent meningitis, suggesting their potential utility as diagnostic indicators for this condition. Additionally, CRP and PCT are proteins with specific functions.. Additionally, CRP and PCT are proteins with specific functions. Following a severe infection, CRP levels rapidly rise, serving as an effective marker for inflammation within the body. Conversely, PCT is primarily secreted by the thyroid gland and typically maintains minimal concentrations under normal physiological conditions. With a short half-life, its level is slightly elevated or not elevated in patients with non-bacterial infections, and it is highly expressed in infectious diseases, making it an ideal indicator for diagnosing infectious diseases [14] [15]. TNF-α serves as both the primary inducer immune inflammatory response, and a pivotal element in the inflammatory »cascade reaction«. It is capable of stimulating the production and release of pro-inflammatory factors such as IL-1β and IL-6, while also promoting the adhesion of inflammatory cells and increasing the permeability of the blood-brain barrier [16]. IL-1β is an inflammatory factor that is closely implicated in diversified pathological injuries in the body. It is the way IL-1β exists in the brain tissue. When the body develops epilepsy due to brain injury, craniocerebral injury, intracranial infection, or other pathologies, its level is clearly elevated [17]. IL-6 is a kind of pro-inflammatory factor that can facilitate the activation of matrix protein metalloenzymes and damage the blood-brain barrier [18]. Therefore, CRP, TNF-α, IL-6, PCT, and IL-1β can be applied as one of the crucial indicators to evaluate the curative effect of refractory purulent meningitis in children. The results of this study demonstrated that levels of CRP, TNF-α, IL-6, PCT, and IL-1β were significantly higher in both the cerebrospinal fluid and blood of the dexamethasone group compared to the standard care and control groups. Meanwhile, these factors were also elevated in the control group compared to the healthy group, which aligns with previous research and indicates a clear elevation of that CRP, TNF-α, IL-6, PCT, and IL-1β in children with refractory purulent meningitis.

Bedetti [19] and Keus *et al.* [20] report that hs- CRP, TNF-α, IL-6 and PCT in the serum and cerebrospinal fluid of neonatal purulent meningitis are elevated and closely linked with the prognosis of children. The findings of this study demonstrate that CRP, TNF-α, IL-6, PCT, IL-1β serve as significant predictors for evaluating the effectiveness of dexamethasone treatment in children diagnosed with refractory purulent meningitis. Moreover, this study also examines the efficacy of CRP, TNF-α, IL-6, PCT, and IL-1β in predicting the effectiveness of dexamethasone treatment in children with refractory purulent meningitis. The AUCs of these factors in predicting the efficacy of dexamethasone in children with refractory purulent meningitis were determined to be 0.877, 0.798, 0.765, 0.736, and 0.817, respectively, all of which had good predictive performance. Notably, CRP and IL-1β demonstrated superior predictive performance, suggesting that CRP, TNF-α, IL-6, PCT, and IL-1β can serve as effective indicators for evaluating efficacy of dexamethasone treatment in children with refractory purulent meningitis.

Overall, children with refractory purulent meningitis exhibit a significant upregulation of inflammation factors, which can serve as reliable indicators for assessing the effectiveness of dexamethasone treatment in this population. However, this study still has the following shortcomings: (1) The children included in this study were all children with refractory purulent meningitis who were admitted to Qingtian County People's Hospital of Lishui City. The sample size is small, and the follow-up time is short. In the later stage, other hospitals should be combined to expand the sample and extend follow-up time; (2) Due to the limitations of the age of the subjects and the wishes of the parents, sampling was difficult, and the cerebrospinal fluid was not detected. It is hoped that these aspects can be noticed at a the later stage to further confirm the results of this study.

## Dodatak

### Acknowledgments

Not applicable.

### Funding

Not applicable.

### Conflict of interest statement

All the authors declare that they have no conflict of interest in this work.
